# Rapid and Robust Multi-Phenotypic Assay System for ALS Using Human iPS Cells with Mutations in Causative Genes

**DOI:** 10.3390/ijms24086987

**Published:** 2023-04-10

**Authors:** Tosho Kondo, Ihori Ebinuma, Hirotaka Tanaka, Yukitoshi Nishikawa, Takaki Komiya, Mitsuru Ishikawa, Hideyuki Okano

**Affiliations:** 1 Department of Physiology, Keio University School of Medicine, Tokyo 160-8582, Japan; 2 Research Center of Neurology, Ono Pharmaceutical Co., Ltd., Osaka 618-8585, Japan; 3 Department of Clinical Regenerative Medicine, Fujita Health University School of Medicine, 1-98, Dengakugakubo, Kutsukake-cho, Toyoake 470-1192, Japan; 4 International Center for Brain Science, Fujita Health University, 1-98, Dengakugakubo, Kutsukake-cho, Toyoake 470-1192, Japan

**Keywords:** amyotrophic lateral sclerosis (ALS), induced pluripotent stem cells (iPSCs), motor neurons, protein accumulation, hyperexcitation, autophagy, degenerative motor neuron diseases

## Abstract

Amyotrophic lateral sclerosis (ALS) is a major life-threatening disease caused by motor neuron degeneration. More effective treatments through drug discovery are urgently needed. Here, we established an effective high-throughput screening system using induced pluripotent stem cells (iPSCs). Using a Tet-On-dependent transcription factor expression system carried on the *PiggyBac* vector, motor neurons were efficiently and rapidly generated from iPSCs by a single-step induction method. Induced iPSC transcripts displayed characteristics similar to those of spinal cord neurons. iPSC-generated motor neurons carried a mutation in *fused in sarcoma* (*FUS*) and *superoxide dismutase 1* (*SOD1*) genes and had abnormal protein accumulation corresponding to each mutation. Calcium imaging and multiple electrode array (MEA) recordings demonstrated that ALS neurons were abnormally hyperexcitable. Noticeably, protein accumulation and hyperexcitability were ameliorated by treatment with rapamycin (mTOR inhibitor) and retigabine (Kv7 channel activator), respectively. Furthermore, rapamycin suppressed ALS neuronal death and hyperexcitability, suggesting that protein aggregate clearance through the activation of autophagy effectively normalized activity and improved neuronal survival. Our culture system reproduced several ALS phenotypes, including protein accumulation, hyperexcitability, and neuronal death. This rapid and robust phenotypic screening system will likely facilitate the discovery of novel ALS therapeutics and stratified and personalized medicine for sporadic motor neuron diseases.

## 1. Introduction

Neurodegenerative diseases such as Alzheimer’s disease, Parkinson’s disease, and amyotrophic lateral sclerosis (ALS) are caused by various mechanisms, such as abnormal protein aggregation, oxidative stress, and abnormal neuronal hyperactivity, for which more effective treatments are desired [1]. Multiple pharmaceutical companies and research groups across the world are aiming to identify effective drug candidates to treat neurodegenerative diseases. However, the development of drugs that show a fundamental curative effect has not occurred. One of the main reasons for this is that no single animal or cellular model provides a complete picture of the disease and often does not reproduce the pathology of human patients [2]. Furthermore, it is difficult to perform direct intervention experiments on living human brain and spinal cord tissue. This problem has led to a focus on disease-specific human cells. For this reason, induced pluripotent stem cell (iPSC) models have become increasingly important in ALS research because they provide a valuable tool to study the cellular and molecular mechanisms underlying this complex disease. By using iPSCs, researchers can generate motor neurons that are affected by ALS and study them in the laboratory, which can provide insights into the disease’s underlying mechanisms and potential treatments [3].

Recent advances in the induction of neuronal differentiation from pluripotent stem cells (PSCs) have led researchers to generate region-specific neurons and brain organoids [4,5] and to establish culture methods that more closely mimic physiological conditions through the use of microfluidic devices and co-culture with glial cells, allowing the study of axonal function and non-cell autonomic effects [6]. In vitro neuronal differentiation methods generally induce PSCs to differentiate into neuroectoderm by inhibiting SMAD signaling, followed by patterning by controlling the concentration of morphogens and compounds that activate or inhibit intracellular signaling [7,8,9,10,11]. This induction method is a powerful tool for studying the pathogenesis of region-specific neuronal diseases and has previously been used for disease modeling and drug discovery, including ALS [12,13,14,15,16]. Previously, we evaluated the efficacy of FDA-approved drugs using motor neurons that were derived from ALS patient-derived iPSCs and found ropinirole, which is a dopamine D2 receptor agonist to treat Parkinson’s disease, to be a promising therapeutic compound [17]. Such induction methods are excellent techniques for mimicking neural development. However, they are often time-consuming and require many complex experimental manipulations, resulting in poor reproducibility of experimental results. Forced expression of Neurogenin2 (NEUROG2/Ngn2), one of the basic helix-loop-helix type pro-neuronal factors, is another method that has been successfully used to generate neurons from PSCs [18]. This method produces highly purified neurons in a short timeperiod. However, only a few drugs have been screened using this technology.

In this study, we optimized the use of three transcription factors, namely NEUROG2, LIM homeobox protein 3 (LHX3), and ISL1 transcription factor, LIM/homeodomain (ISL1), collectively known as NIL factors, which have been previously used for neuronal differentiation [19,20,21]. Briefly, ISL1 and LHX3, whose expression is upregulated early in spinal motor neuron differentiation, have the ability to cooperate with each other to activate the transcriptional network of various spinal motor neuron marker gene clusters, including MNX1 (HB9) [22]. Using these optimized NIL factors, we induced differentiation of lower motor neurons more rapidly and efficiently and developed them into a robust high-throughput screening system. Using iPSCs carrying a mutation in *superoxide dismutase 1* (*SOD1*) [17] and *fused in sarcoma* (*FUS*) [12], which were used in our previous report, we confirmed that induced motor neurons also showed an accumulation of aggregated proteins corresponding to each mutation, respectively. Functionally, both *SOD1*-ALS and *FUS*-ALS motor neurons exhibited hyperexcitability, as reported previously [13]. Thus, our optimized differentiation induction system is expected to produce rapid and robust phenotypic expression, which will accelerate the screening of drugs that may suppress cellular dysfunction and cell death.

## 2. Results

### 2.1. Motor Neurons Were Rapidly and Efficiently Generated from iPSCs

**Figure 1:**
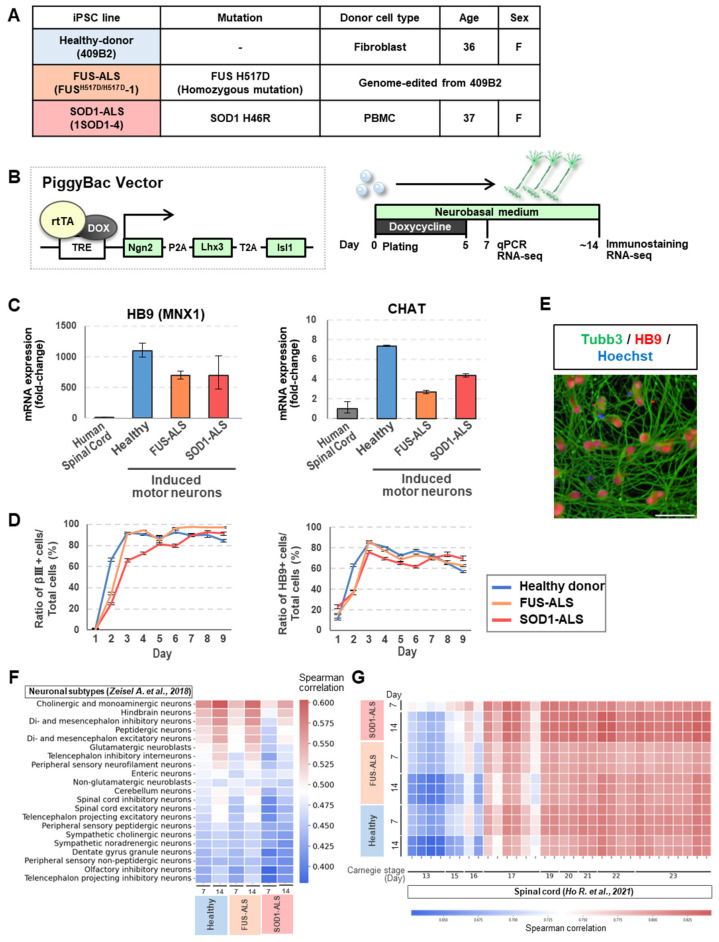
Generation of motor neurons from iPSCs. (**A**) Information about the iPSCs used in this study. (**B**) Experimental design of neuronal differentiation and analysis; (**C**) mRNA expression levels of motor neuron markers on day 7 were determined by qPCR. Each value represents the mean ± SEM (*n* = 3). (**D**) Induction efficiency for motor neurons was evaluated for days 1–9. Each value represents mean ± SEM (*n* = 16). (**E**) A representative image of healthy donor-derived motor neurons on day 7 immunostained for Tubb3 (green), HB9 (red), and Hoechst 33342 (blue). The scale bar represents 50 μm. (**F**) The transcriptome of induced motor neurons on days 7 and 14 were compared to each cluster of annotated neurons from the adult mouse scRNA-seq dataset [25]. (**G**) The transcriptome of induced motor neurons on days 7 and 14 was compared to that of the human spinal cord at each Carnegie stage [26].

In this study, we established a single-step method to induce motor neurons from laminin511-E8 fragment-based feeder-free iPSCs and reproduce the disease phenotype observed in a conventional assay system. We previously established an assay system for detecting protein accumulation, neurite retraction, and neuronal cell death in ALS patient-derived iPSCs, including three cell lines [17]. The three cell lines were 409B2, FUS^H517D/H517D^-1, and 1SOD1-4, which were used as healthy controls, genome-edited 409B2 cell line with *FUS* homozygous mutation, and *SOD1*-mutated patient lines, respectively (Figure 1A). Previous studies have shown that lower motor neurons can be induced from PSCs by overexpressing three transcription factors called NIL factors: NEUROG2/Ngn2, LHX3/Lhx3, and ISL1/Islet1 [19,20,21]. In this study, we engineered NILs that were expressed in a Tet-On-driven manner using *PiggyBac* vectors. Although these cells proliferated in large numbers after drug selection with hygromycin and G418 and can be cryopreserved, even after thawing, they were readily differentiated into neurons by doxycycline treatment (Figure 1B). First, we confirmed that they were motor neurons by detecting the mRNA expression of the motor neuron markers HB9/MNX1 and CHAT on day 7 using qPCR. These induced motor neurons showed sufficient expression of HB9 and CHAT compared to human spinal cord tissue, which was used as a positive control, despite containing a variety of cells, including interneurons, glial cells, and blood vessels (Figure 1C). Subsequently, we examined the protein expression of motor neuron markers over the culture period. After starting doxycycline treatment on day 0, fixed samples were prepared daily from days 1 to 9 for immunostaining to quantify HB9, CHAT and βIII-tubulin in each population. The percentage of HB9- and βIII-tubulin-positive cells increased rapidly as early as day 3 (Figure 1D), and a neuron-like morphology was observed by day 7 (Figure 1E). In addition, we also identified a staining signal for CHAT as a marker of mature motor neurons, whereas there was almost no signal under conditions of glutamatergic neuron induction by NEUROG2 alone (Figure S1). Therefore, the disease phenotypic assays were performed after day 7. We also analyzed the transcriptome of the induced motor neurons on days 7 and 14 using RNA sequencing (RNA-seq). To assess the developmental and functional status of these cultures, we correlated their expression profiles to neuronal clusters identified in single-cell RNA-seq (scRNA-seq) analysis of the mouse adult nervous system [23] and to the human fetal hindbrain and spinal cord tissues ranging from Carnegie stages 13 to 23 [24]. On day 7, the induced neurons showed the highest correlation with cholinergic neurons of the central and peripheral nervous system in mice, and this correlation became more pronounced on day 14 (Figure 1F). By day 14, they exhibited transcriptional states that most closely resembled those of the human fetal spinal cord and hindbrain tissue at the Carnegie stage 22 or later (Figure 1G and Figure S2).

### 2.2. Protein Accumulation Corresponding to Gene Mutations Were Observed in iPSC-Derived Neurons

**Figure 2:**
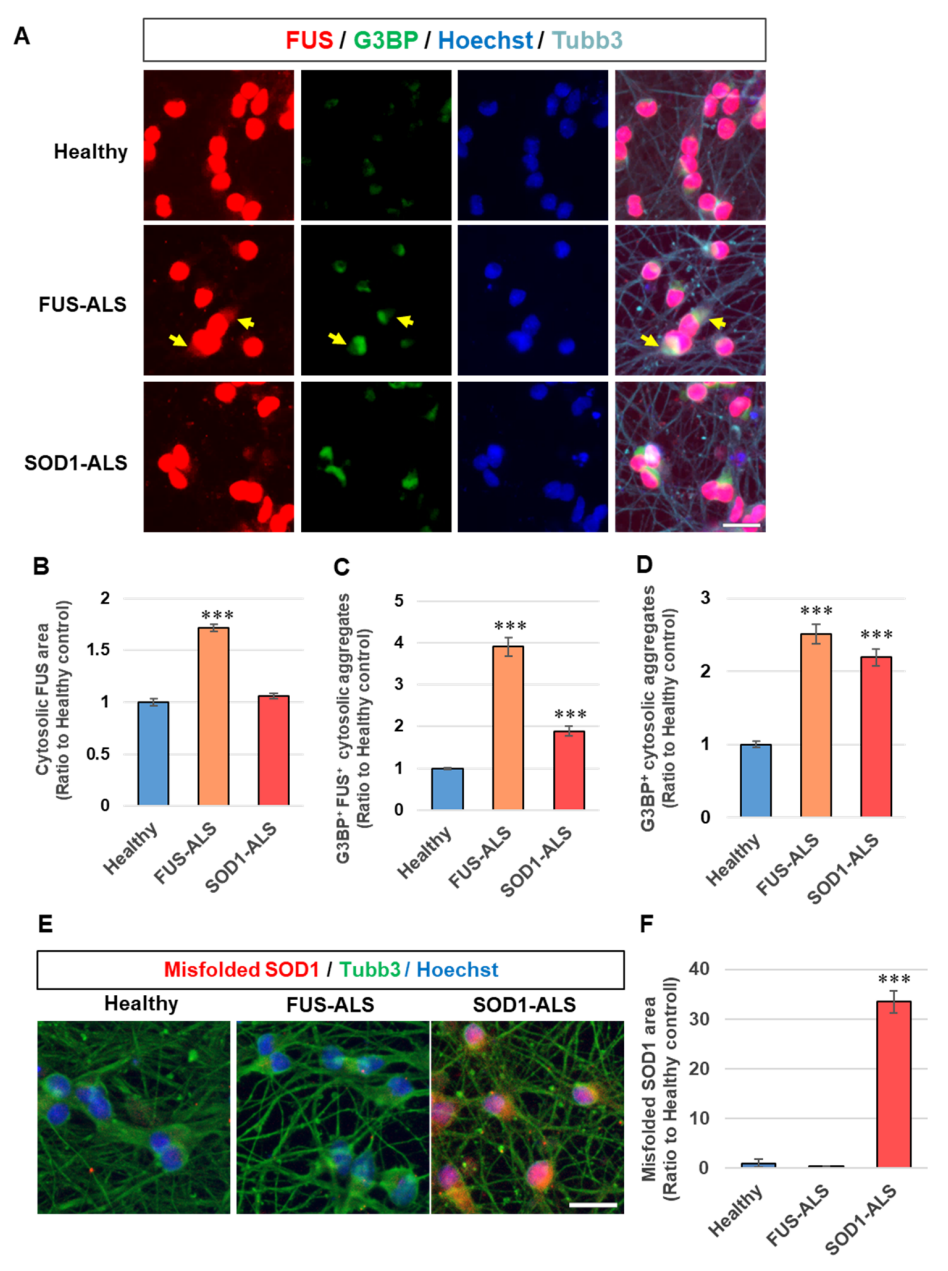
Evaluation of protein accumulation in the ALS neurons. (**A**) Representative images of iPSC-derived motor neurons immunostained for G3BP (green), FUS (red), Tubb3 (cyan) and Hoechst 33342 (blue) on day 14. Yellow arrowheads indicate the cytosolic FUS+ region. The scale bar represents 50 μm. Quantification of: (**B**) cytosolic FUS+ area; (**C**) FUS + G3BP+ cytosolic aggregates; and (**D**) G3BP+ cytosolic aggregates per cell. Each readout of *FUS*-ALS and *SOD1*-ALS neurons was normalized to that of the healthy control (*n* = 6, mean ± SEM). (**E**) Representative image of iPSC-derived motor neurons immunostained for Tubb3 (green), misfolded SOD1 (red), and Hoechst 33342 (blue) on day 14. The scale bar represents 50 μm. (**F**) Quantification of misfolded SOD1 area per cell. The misfolded SOD1 area of *FUS*- and *SOD1*-ALS neurons was normalized to that of the healthy control (*n* = 6, mean ± SEM). ***: *p* < 0.001 vs. healthy control (*t*-test).

We previously observed the formation of cytosolic FUS+ stress granules and SOD1 aggregates in *FUS*-ALS and *SOD1*-ALS motor neurons, respectively [17]. In this study, we tested whether the accumulation of each protein could be reproduced using doxycycline induction. FUS and G3BP, Ras GTPase-activating protein-binding protein, which is widely used as a marker for stress granules, were immunostained on day 14. The cytosolic FUS+ region increased in *FUS*-ALS neurons, which was not observed in healthy or *SOD1*-ALS neurons (Figure 2A,B), suggesting that cytoplasmic mislocalization of FUS is a specific phenotype of the *FUS* mutation. Additionally, G3BP+ aggregates colocalized with cytosolic FUS in *FUS*-ALS neurons (Figure 2A,C). In contrast, G3BP+ cytosolic aggregates significantly accumulated in both *FUS*- and *SOD1*-ALS neurons (Figure 2A,D). SOD1 is not a constituent protein of stress granules, but mutant SOD1 binds to G3BP1 in an RNA-independent manner and affects aberrant stress granule formation in spinal motor neurons [25]. Thus, we hypothesized that increased stress granule formation is a common phenotype in *FUS*- and *SOD1*-ALS. Next, immunostaining for misfolded SOD1 was performed on day 14. Misfolded SOD1 significantly accumulated in *SOD1*-ALS neurons, which was not observed in healthy donors and *FUS*-ALS neurons (Figure 2E,F). Based on these results, we concluded that the pathology of each ALS mutation was recapitulated in our induced motor neurons within two weeks of culture.

### 2.3. Protein Accumulation in Motor Neurons Was Suppressed by the Activation of Autophagy 

**Figure 3:**
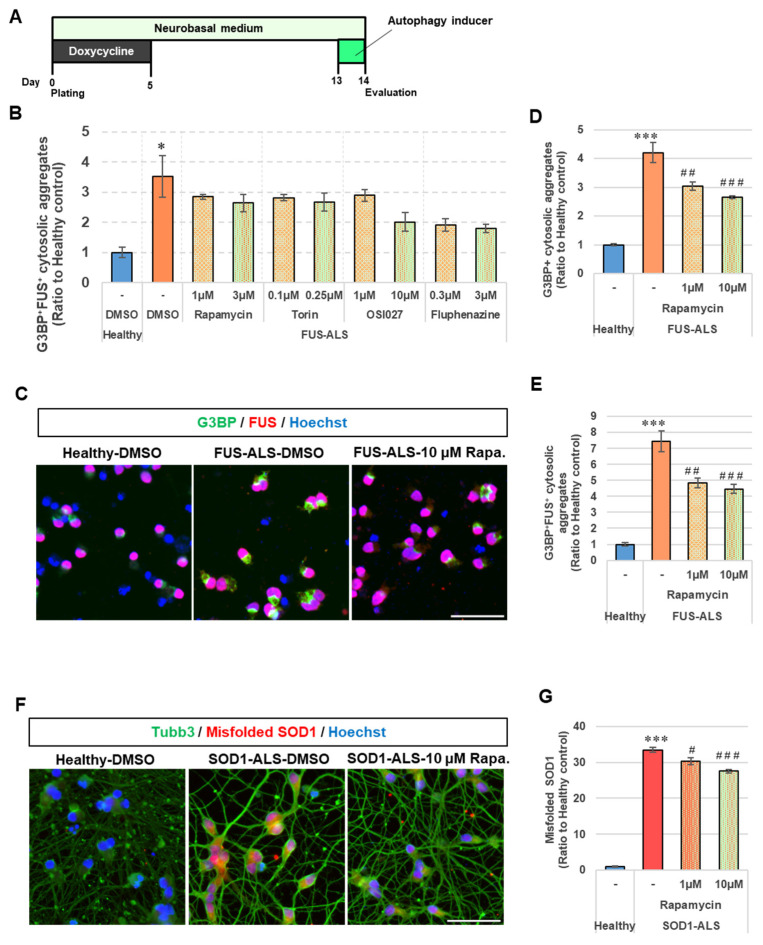
Effect of autophagy inducers on protein accumulation in ALS neurons. (**A**) Overview of the evaluation system for autophagy inducers. (**B**) The effect of autophagy inducers on FUS+ stress granules in *FUS*-ALS neurons was evaluated. Accumulation of FUS + G3BP+ cytosolic aggregates in *FUS*-ALS neurons was normalized to that of healthy control (*n* = 3, mean ± SEM). (**C**) Representative images of iPSC-derived motor neurons immunostained for G3BP (green), FUS (red), and Hoechst 33342 (blue). The scale bar represents 50 μm. The effect of rapamycin on (**D**) G3BP+; and (**E**) FUS + G3BP+ stress granule formation was evaluated. Accumulation levels of stress granules in *FUS*-ALS neurons were normalized to that of healthy controls (*n* = 5, mean ± SEM). (**F**) Representative images of iPSC-derived motor neurons immunostained for Tubb3 (green), misfolded SOD1 (red), and Hoechst 33342 (blue). The scale bar represents 50 μm. (**G**) The effect of rapamycin on SOD1 accumulation was evaluated. Accumulation levels of SOD1 in *SOD1*-ALS neurons were normalized to that of healthy controls. (*n* = 5, mean ± SEM). *: *p* < 0.05 vs. healthy control (*t*-test), ***: *p* < 0.001 vs. healthy control (*t*-test); #: *p* < 0.05 vs. vehicle control (Dunnett test), ##: *p* < 0.01 vs. vehicle control (Dunnett test), ###: *p* < 0.001 vs. vehicle control (Dunnett test).

To validate our assay system, we investigated whether the accumulation of aggregated proteins in ALS neurons could be suppressed by autophagy activation. First, we evaluated the effects of several autophagy inducers, specifically, rapamycin [26], torin, OSI-027 F [27], and fluphenazine [28]) on stress granule formation in *FUS*-ALS neurons (Figure 3A). FUS + G3BP+ cytosolic stress granules in *FUS*-ALS neurons tended to decrease upon treatment with all compounds (Figure 3B). Since autophagy inducers generally act on *FUS*-ALS neurons, the effect of rapamycin, a well-studied autophagy inducer, was evaluated in detail. We found that both G3BP+ and FUS + G3BP+ cytosolic aggregates increased in *FUS*-ALS neurons and that aggregate formation was significantly suppressed by rapamycin treatment in a dose-dependent manner (Figure 3C–E). Next, we evaluated the effects of rapamycin on *SOD1*-ALS neurons. Rapamycin significantly suppressed the accumulation of misfolded SOD1 in *SOD1*-ALS neurons in the same concentration-dependent manner as aggregate formation in FUS-ALS neurons (Figure 3F,G). Based on these data, we concluded that the accumulation of aggregated proteins observed in ALS neurons represents the ALS pathology observed in human patients, and that autophagy enhancers are likely to have therapeutic potential for ALS. 

### 2.4. Induced Motor Neurons Have Electrophysiological Function and the ALS Motor Neurons Show Hyperexcitability

**Figure 4:**
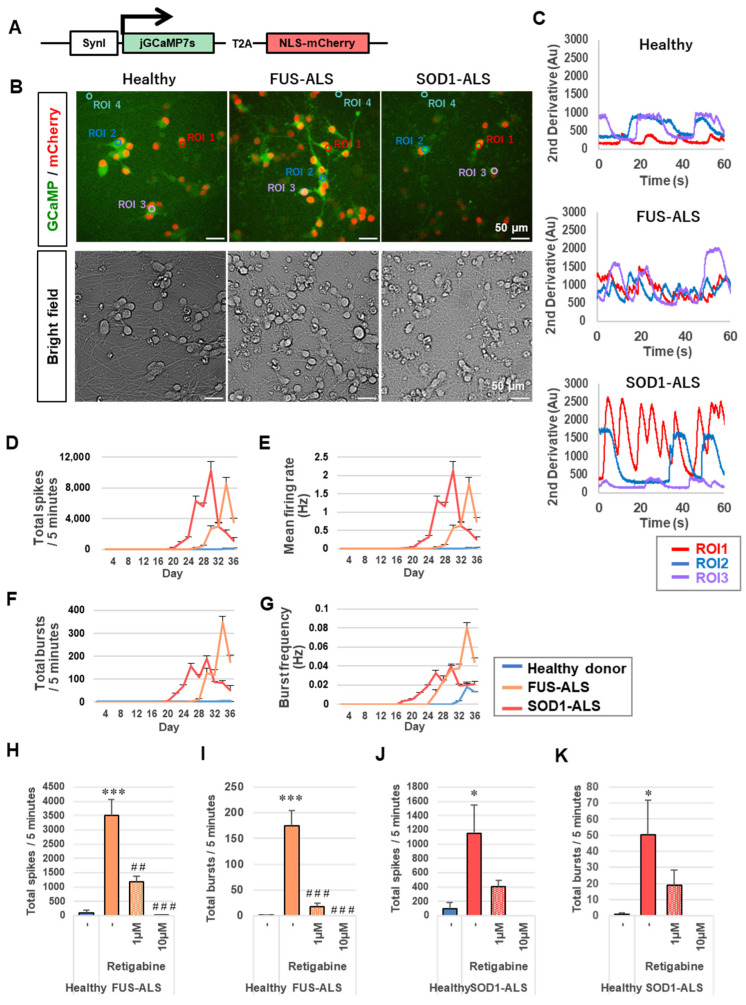
Evaluation of neuronal activity and hyperexcitation in ALS using iPSC-derived motor neurons. (**A**) Vector design for neuronal expression of GCaMP and mCherry in the nucleus. (**B**) Representative GCaMP, mCherry, and brightfield images of the induced motor neuron on day 14. ROIs (ROI1-3) are depicted in (**C**) as time-dependent changes in GFP fluorescence intensity. Scale bar: 50 μm. From days 4–36: (**D**) the number of spikes; (**E**) mean firing rate; (**F**) the number of bursts; and (**G**) burst frequency was evaluated. Each value represents mean ± SEM (Healthy: *n* = 11, *FUS*-ALS: *n* = 11, *SOD1*-ALS: *n* = 10, mean ± SEM). The effect of retigabine on the increase in (**H**) the number of spikes; (**I**) bursts in *FUS*-ALS and the number of (**J**) spikes; and (**K**) bursts in *SOD1*-ALS were evaluated on day 36 (healthy: *n* = 5, FUS-DMSO: *n* = 4, FUS-1 μmol L^−1^ retigabine: *n* = 3, FUS-10 μmol L^−1^ retigabine: *n* = 4, SOD1-DMSO: *n* = 4, SOD1-1 μmol L^−1^ retigabine: *n* = 3, SOD1-10 μmol L^−1^ retigabine: *n* = 3, mean ± SEM). *: *p* < 0.05 vs. healthy control (*t*-test), ***: *p* < 0.001 vs. healthy control (*t*-test); ##: *p* < 0.01 vs. vehicle control (Dunnett test), ###: *p* < 0.001 vs. vehicle control (Dunnett test).

It has been suggested that increased cytotoxicity due to hyperexcitation may play a role in the pathogenesis of ALS. To determine whether the induced motor neurons were mature enough to show electrical activity, we first performed calcium imaging. jGCaMP7s and nuclear localization signal (NLS)-mCherry, expressed downstream of the Synapsin I (SynI) promoter (Figure 4A), were transfected into iPSCs to measure neuron-specific calcium concentrations. Regions of interest, including the cell body (ROI1–3) and cell-free region (ROI4) for background intensity calculation, were defined from the induced neurons on day 14 in each field of view (Figure 4B). All mCherry+ cells showed signal oscillation of GCaMP, which indicates spontaneous oscillations of intracellular calcium levels, and the amplitude-frequency tended to be higher in ALS neurons. (Figure 4C). To verify the electrophysiological function of the induced motor neurons in another assay system, we attempted to detect neuronal activity using multiple electrode array (MEA) recordings. iPSCs were seeded onto a plate with 16 electrodes per well. Neuronal induction was performed as described above (Section 2.1, Section 2.2 and Section 2.3), and MEA recordings were measured every two days until day 36. As the spikes of induced motor neurons were detected in all three iPSC lines around day 28, the differentiation of iPSCs into electrically mature neurons was considered successful. In addition, we found that *FUS*-ALS and *SOD1*-ALS neurons had significantly higher spontaneous firing rates than healthy neurons (Figure 4D–G). To validate the hyperexcitability observed in ALS neurons, we evaluated the effects of retigabine as a positive control compound [13]. On day 36, just before the end of the experiment, treatment with retigabine for 3 h inhibited hyperexcitation in ALS neurons in a dose-dependent manner (Figure 4H–K). Our induction method is also useful for evaluating neuronal hyperactivity.

### 2.5. Hyperexcitability of ALS Motor Neurons Was Suppressed by Activation of Autophagy

**Figure 5:**
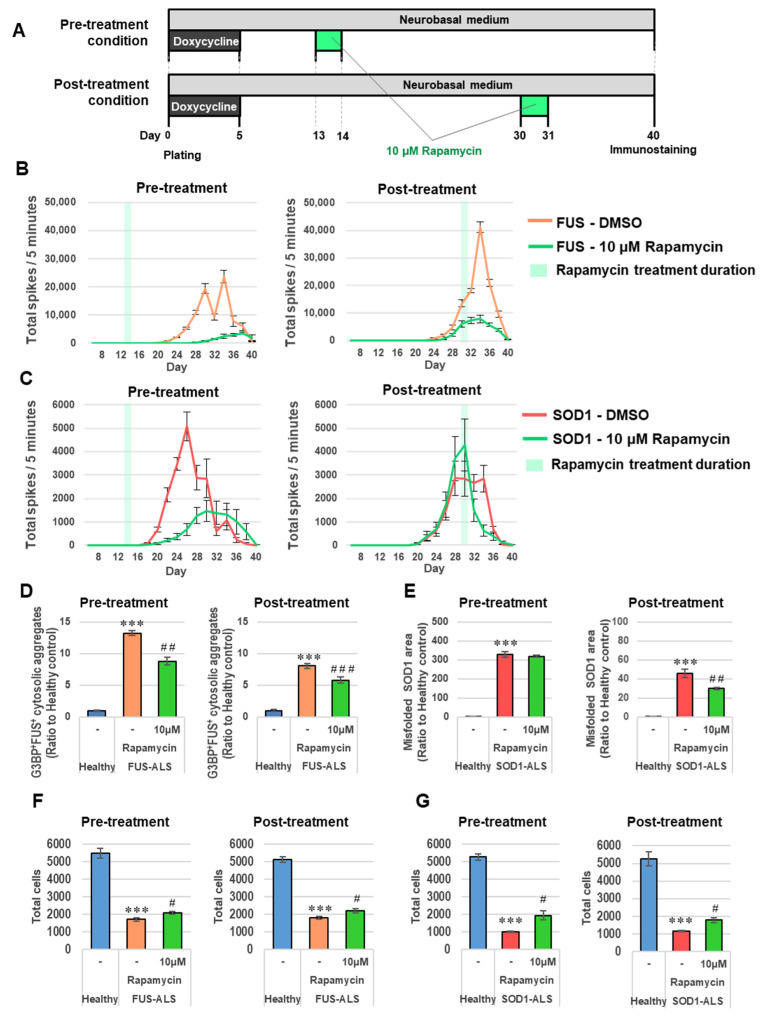
Effect of rapamycin on hyperexcitability in ALS neurons. (**A**) Overview of the scoring system for the effect of rapamycin on hyperexcitability in ALS-neurons. The number of spikes in (**B**) *FUS*-ALS; and (**C**) *SOD1*-ALS following pre-and post-treatment of rapamycin were evaluated from day 6 to 40. Each value represents the mean ± SEM (*n* = 4). The effect of rapamycin on (**D**) FUS stress granule formation in *FUS*-ALS; and (**E**) the accumulation of misfolded SOD1 in *SOD1*-ALS were evaluated on day 40. Each value was normalized to that of the healthy control (*n* = 4, mean ± SEM). The effect of rapamycin on the cell counts of (**F**) FUS-ALS; and (**G**) SOD1-ALS were evaluated on day 40. Each value represents mean ± SEM (*n* = 4). ***: *p* < 0.001 vs. healthy control (*t*-test); #: *p* < 0.05; ##: *p* < 0.01; ###: *p* < 0.001 vs. vehicle control (Dunnett test).

Although abnormalities in protein accumulation and neuronal activity are associated with ALS pathology, the relationship between these two phenotypes remains unclear. Next, we investigated whether hyperexcitation could be ameliorated when protein accumulation was suppressed by autophagy activation. Since a significant increase in spontaneous firing in ALS neurons was observed by MEA at around day 30 (Figure 4D–G), we recorded neuronal activity when rapamycin was treated from day 13 to 14 (pre-treatment) or from day 30 to 31 (post-treatment) (Figure 5A). Both pre-treatment and post-treatment with rapamycin suppressed the number of spikes in *FUS*-ALS and *SOD1*-ALS motor neurons, although the peak of hyperexcitation overlapped with the timing of administration on day 30, making it difficult to determine the efficacy of treatment with rapamycin in *SOD1*-ALS neurons (Figure 5B,C). To confirm whether the accumulation of aggregated proteins was suppressed by each rapamycin treatment, the high-content imaging analysis described above (Section 2.2 and Section 2.3) was performed on day 40, the last day of MEA recording. In *FUS*-ALS, both pre- and post-treatment with rapamycin suppressed the formation of FUS + G3BP+ cytosolic stress granules (Figure 5D). Further, rapamycin treatment also decreased the accumulation of misfolded SOD1, although its efficacy was not observed in the pre-treatment condition (Figure 5E). To confirm that the inhibitory effect of rapamycin on ALS neuronal hyperexcitability was not due to cytotoxicity, cell counts were assessed on day 40. The number of cells decreased in *FUS*-ALS and *SOD1*-ALS neurons compared to that in the healthy control, and rapamycin restored the number of cells (Figure 5F,G). These results indicate that rapamycin ameliorates abnormal neuronal hyperactivity without causing cytotoxicity and has a protective effect against neuronal cell death.

## 3. Discussion

The application of iPSC-based neurological disease modeling for drug discovery has recently attracted the attention of researchers [1]. Noticeably, compounds found by screening using patient-derived iPSCs have had encouraging results in ALS clinical trials [29,30], suggesting it to be a promising strategy for drug development. Many compound screening studies have used motor neurons differentiated from ALS patient-derived iPS cells [31]. Previously, we identified ropinirole, a dopamine D2 receptor agonist, as a therapeutic agent that ameliorates several in vitro phenotypes associated with ALS [17]. This study employed a screening system that made use of neurons that underwent differentiation in response to dual inhibition of SMAD; however, the experimental procedure is complex and requires a long culture period. Another neuronal differentiation method uses PSCs to generate spinal motor neurons by forced expression of NEUROG2, ISL1, and LHX3 (NIL factors) [19] and has facilitated the discovery of therapeutic candidates such as Bosutinib (Src/c-Abl inhibitor) [20] and Apilimod (PIKFYVE inhibitor) [21]. While the NIL method is a simple experimental manipulation and can be easily implemented in a short culture period, the resulting motor neurons are not necessarily generated according to physiological developmental mechanisms, and consequently, their ability to facilitate screening of therapeutic agents at an early stage of culture remains questionable.

We sought to optimize a rapid, one-step, multi-phenotype screening system by introducing NIL factors. In this induction system, motor neuron differentiation is initiated by simply switching from the iPSC medium to the Neurobasal medium containing doxycycline during iPSC passaging, demonstrating its convenience and ease of use. Importantly, this induction method rapidly yielded a purified population of motor neurons, regardless of the cell lineage. In addition, the transcriptome of motor neurons was most similar to that of human and mouse spinal cord tissues, suggesting that this method may be useful for the analysis of motor neuron diseases.

Accumulation of aggregated proteins is an important phenotype of ALS. Using an immunoprecipitation approach, the positive effects of bosutinib on the dissociation of SOD1 and TDP-43 aggregation have been identified [20]. In our study, using a high-content cell imager as a high-throughput method, the removal of FUS and SOD1 accumulation by rapamycin was easily detected. Aberrant excitation of motor neurons is reported to be a key event leading to neuronal death in ALS. Retigabine has already been identified as a therapeutic agent that inhibits the hyperexcitability of motor neurons in vitro [13]. Further, iPSC-derived neurons were also seeded into primary mouse cortical glia, which can increase the firing rates [32]. Owing to the simplicity of our induction system, the hyperexcitability of ALS neurons was clearly detected without glial feeder cells, suggesting that our method could detect abnormal neuronal activity faster than other methods. We also noticed that rapamycin inhibits neuronal hyperexcitation in ALS. These results suggest that the removal of aggregated proteins through the activation of autophagy leads to an improvement in neuronal activity in ALS.

The cell culture system used in this study allowed us to rapidly analyze pathological protein aggregation and hyperexcitation in iPSC-derived motor neurons. However, as reported by multiple iPSC studies, it is unclear why the disease phenotype is observed early. Further, since this study focused only on neurons, our approach may be less useful for studying pathological conditions significantly affected by glial cell abnormalities. Nonetheless, in our culture system, the motor neuron ALS phenotype could be rapidly generated, with high purity, and in large quantities. Moreover, this method remains robust and shows high-quality differentiation with little difference between the cell lines. Thus, our method is well-suited for large-scale drug screening. We anticipate that this assay system will be applicable to the study of sporadic ALS, which requires a large number of patients. We hope to identify effective drug candidates for ALS through multi-phenotype screening using this assay system which can hopefully be used by ALS patients in the near future.

## 4. Materials and Methods

### 4.1. Human iPSC Culture

409B2 [33] was used as healthy control human iPSC line. FUS^H517D/H517D^-1 [12] was used as *FUS* mutated isogenic line. 1SOD1-4 [17] was used as *SOD1* mutant patient line. iPSCs were maintained in StemFit/AK02N medium (Ajinomoto, Chuo-ku, Tokyo, Japan) without feeder cells. Cells were passaged with 0.5 × TrypLE Select (Thermo Fisher Scientific, Waltham, MA, USA) in PBS (−) and seeded at a density of 1.0 × 10^4^ cells/well on iMatrix 511/Laminin-511 E8 (Nippi, Adachi-ku, Tokyo, Japan)-treated 6-well plates. A total of 10 μM Y27632 (Nacalai, Nakagyo-ku, Kyoto, Japan) was added only for the first day. The culture media were changed every other day.

### 4.2. PiggyBac Vector Transfection

According to the previous study [34,35], tet-on inducible iPSCs of NIL factors were established using the following vectors: PB-Neo-TRE3G-hNEUROG2-P2A-hLHX3-T2A-hISL1, pCMV-HyPBase-PGK-Puro and PB-CAG-rtTA3G-IH. These vectors were co-transfected into dissociated iPSCs using Gene Juice Transfection Reagent (Merck, Darmstadt, Land Hessen, Germany), as described previously. Transfectants were cultured in StemFit/AK02N containing 20 μM Y27632 (Wako, Chuo-ku, Tokyo, Japan), 50–150 μg/mL hygromycin (Nacalai), and 100–1000 μg/mL G418 (Nacalai) on iMatrix 511-treated 6-well plate. All surviving colonies were expanded without selection for a single clone. They were stored appropriately by StemCellBanker (ZENOAQ, Fukushima, Japan) and used for this study.

### 4.3. Neuronal Differentiation from iPSCs

Motor neurons were generated from feeder-free iPSCs by transient expression of NIL factors using the tet-on inducible expression system. At the passage, dissociated iPSCs were seeded onto poly-L-ornithine (Merck) and Matrigel (Corning, Steuben County, NY, USA) coated culture plates and cultured in neuronal medium with the following composition: Neurobasal Plus medium (Thermo Fisher Scientific) containing 2% B27 Plus supplement (Thermo Fisher Scientific), 1% Culture One supplement (Thermo Fisher Scientific), 1% Glutamax (Thermo Fisher Scientific), 200 μM L-ascorbic acid (Sigma), 200 μM dbcAMP (Nacalai), 20 ng/mL BDNF (Alomone labs), 20 ng/mL GDNF (Alomone labs), and 20 ng/mL NT-3 (Alomone labs). 20 μM Y27632, 1 μg/mL doxycycline (Wako), 1 μM retinoic acid (Merck), 1 μM purmorphamine (Merck) and 0.5 μg/mL iMatrix-511 silk were added only for 5 days. Half of the medium was changed every 3 or 4 days after day 5.

### 4.4. Quantitative Reverse-Transcription PCR (qRT-PCR)

Total RNA was extracted using an RNeasy mini kit (Qiagen, Hilden, North Rhine-Westphalia, Germany) according to the manufacturer’s protocol. cDNAs were synthesized using an iScript cDNA synthesis kit (Bio-Rad, Hercules, CA, USA). The qRT-PCR was performed with SYBR Premix ExTaqII (TaKaRa Bio, Kusatsu, Shiga, Japan) using the ViiA7 real-time PCR system (Thermo Fisher Scientific). The values were normalized to ActB levels. Data were analyzed by the comparative (^ΔΔCt^) method. Sequences of primer are listed in Supplemental Table S1.

### 4.5. Transcriptome Analysis

Total RNA was extracted using an RNeasy mini kit (Qiagen) according to the manufacturer’s protocol. The RNA concentration was measured using NanoDrop (Thermo Fisher Scientific), and the quality of RNA was analyzed using the Agilent 4200 TapeStation (Agilent, Santa Clara, CA, USA), RNA Screen Tape (Agilent), and RNA reagent kit (Agilent), after which the samples were stored at −80 °C. The method of the confirmation of RNA quality was in accordance with the manufacturer’s instructions. The RNA was used to prepare a cDNA library for RNA sequencing. TruSeq Stranded mRNA HT kit (Illumina, San Diego, CA, USA) was used for library preparation. The concentration of the prepared library was measured by Qubit (Thermo Fisher Scientific). The Qubit dsDNA HS Assay Kit (Thermo Fisher Scientific) was used, and the method was performed according to the kit manual. The length of the DNA fragment of the library was analyzed by the Agilent 4200 TapeStation. The DNA fragment length was determined using the D1000 ScreenTape (Agilent) and the D1000 reagent kit (Agilent). Each library was stored at −20 °C. The libraries with confirmed quality were sequenced with pair-end sequencing using the NextSeq 500 System (Illumina) and the NextSeq 500/550 High Output Kit v2 (150 Cycles) (Illumina). The library preprocessing was performed according to the manufacturer’s instructions.

### 4.6. Immunocytochemistry

Cells were fixed with 4% paraformaldehyde (Wako) for 30 min at room temperature and then washed twice with PBS (−). They were then blocked with PBS (−) containing 5% normal goat serum and 0.3% Triton X-100 for 1 h at room temperature and incubated overnight at 4 °C with the primary antibodies described in Supplementary Table S2. Cells were washed twice with PBS (−) and then incubated with secondary antibodies Alexa Fluor 488, Alexa Fluor 555, or Alexa Fluor 647 (1:250; Thermo Fisher Scientific) and Hoechst 33258 (0.5 μg/mL; Sigma-Aldrich) for 1 h at room temperature. After washing with PBS (−), a fluorescence image was obtained using the IN Cell Analyzer 6000 (Cytiva, Shinjuku-ku, Tokyo, Japan). In the quantitative analysis of cytoplasmic FUS, due to the limitation of dynamic range adjustment of the FUS immunostaining signal, we allowed the cell nuclei to be saturated for cytoplasmic signal quantification.

### 4.7. High-Content Analysis

Stained 96 well plates were imaged using the IN Cell Analyzer 6000; a set of 5 × 5 fields was collected from each well using the 10× objective lens. Quantitative analysis was performed using IN Cell Developer Toolbox version 1.9 (GE Healthcare, Chicago, IL, USA). For induction efficiency analysis, the ratio of βIII+ cells/total cells, which is the number of nuclei counted by Hoechst staining, and HB9+ cells/total cells (%) was quantified. For protein accumulation analysis, the sum of cytosolic FUS+ area, G3BP+ aggregates area, FUS + G3BP+ aggregates area, or SOD1+ area/the number of βIII+ cells was quantified. 

### 4.8. Calcium Imaging

iPSCs were infected with Synapsin1:jGCaMP7s-T2A-NLS (nuclear localization signal)-mCherry lentivirus, as established by our previous report [34] 6 days prior to neuronal induction (MOI = 10). Changes in fluorescence intensity were measured using an IX83 inverted microscope (Olympus, Shinjuku-ku Tokyo, Japan) equipped with an Electron Multiplying CCD camera (Hamamatsu Photonics) and a pE-4000 LED illumination system (CoolLED, Andover, Hampshire, UK). We recorded 2000 frames (1 frame: 31–32 ms) per field of view using the stream acquisition mode. CellSens dimension software v4.1.1 (EVIDENT, Shinjuku, Tokyo, Japan) was used for data analysis. Regions of interest (ROIs) were defined for mCherry-positive neurons only. The average fluorescence intensity within each ROI was calculated, and the background intensity of the cell-free region was subtracted to adjust for photobleaching in each image.

### 4.9. Multiple Electrode Array (MEA)

MEA was performed using Maestro systems (Axion Biosystems, Atlanta, GA, USA). Neuronal induction was performed on 48-well MEA plates coated with 0.15% polyethyleneimine and 50× Matrigel. Each well was seeded with 2.0 × 10^5^ cells in 30 μL culture medium. 2 h after seeding, 470 μL of culture medium was added. As well as neuronal induction for other analyses, Y27632, doxycycline, retinoic acid, purmorphamine, and iMatrix-511 silk were added only for 5 days, and half of the medium was changed every 4 days after day 5. Data were acquired at a sampling rate of 12.5 kHz and filtered with a 200–3000 Hz Butterworth bandpass filter. The detection threshold was set to 6 × SD of the baseline electrode noise. Spontaneous activity was recorded at 37 °C for 5 min every 2 days. Single electrode bursts were defined as the number of >5 spikes/100 ms. The number of spikes, mean firing rate (spikes per second), number of bursts, and burst frequency (bursts per second) were measured using the Axion Integrated Studio program (Axion Biosystems).

### 4.10. Drug Treatment

Rapamycin, Torin 1, OSI-027, and fluphenazine dihydrochloride were purchased from Funakoshi (Bunkyo-ku, Tokyo, Japan), Tocris Bioscience (Bristol, UK), MedChemExpress (Monmouth Junction, NJ, USA), and Tronto Research Chemical (North York, ON, Canada), respectively. Retigabine hydrochloride was synthesized and purified by Ono Pharmaceutical Co., Ltd. All drugs were dissolved in dimethyl sulfoxide (DMSO) to prepare 10 mmol/L stock solutions and stored in a freezer at −30 °C. 10 mmol/L stock solutions were further diluted in DMSO to desired concentration. DMSO or compound solutions were diluted with culture medium to a final concentration of 0.1 *w*/*v*%, and cells were treated on the cells on the day of compound administration.

## Data Availability

The data that support this study’s findings are available from the corresponding authors upon reasonable request.

## Supplementary Materials

The following supporting information can be downloaded at: https://www.mdpi.com/article/10.3390/ijms24086987/s1.
