# Supplementary material for: Rapid and Robust Multi-Phenotypic Assay System for ALS Using Human iPS Cells with Mutations in Causative Genes
Source: Int J Mol Sci. 2023 Apr 10;24(8):6987. doi: 10.3390/ijms24086987 (PMC10138792; doi:10.3390/ijms24086987)
Supplement: Supplementary file 1 [file ijms-24-06987-s001.zip › Kondoet al SupTable_Submit_230407.pdf]

Supplementary Table S1

List of primers (related to Figure 1C)

Gene	Forward	Reverse
**ACTB**	CACCATTGGCAATGAGCGGTTC	AGGTCTTTGCGGATGTCCACGT
**HB9 (MNX1)**	GTCCACCGCGGGCATGATCC	TCTTCACCTGGGTCTCGGTGAGC
**CHAT**	GGAGGCGTGGAGCTCAGCGACACC	CGGGGAGCTCGCTGACGGAGTCTG

Supplementary Table S2

List of antibodies (related to Figure1D, 1E, 2, 3, 5D, and 5E)

Protein	Species	Isotype	Source (Cat no.)	Dilution
**βIII-TUBULIN**	Mouse	IgG2b	Merck (T8660)	1 : 1000
**HB9**	Mouse	IgG1	DSHB (81.5C10)	1 : 1000
**FUS**	Rabbit	(Polyclonal)	Bethyl Laboratories (A300-293A)	1 : 1000
**G3BP**	Mouse	IgG1	BD Bioscience (611126)	1 : 500
**Misfolded SOD1**	Mouse	IgG1	MediMabs (B8H10)	1 : 100
