# Supplementary figures and images for: Rapid and Robust Multi-Phenotypic Assay System for ALS Using Human iPS Cells with Mutations in Causative Genes
Source: Int J Mol Sci. 2023 Apr 10;24(8):6987. doi: 10.3390/ijms24086987 (PMC10138792; doi:10.3390/ijms24086987)

Supplementary Figure S1

Tubb3 / CHAT / Hoechst

NEUROG2

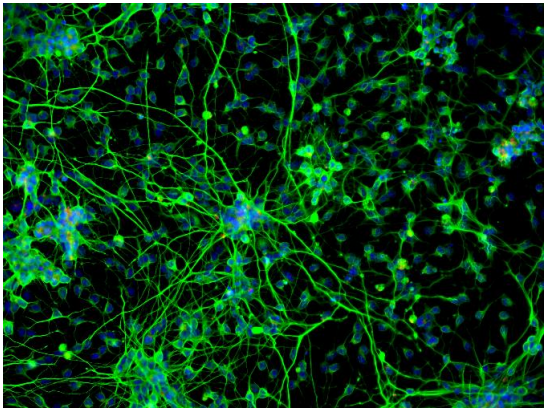

NEUROG2, LHX3, ISL1

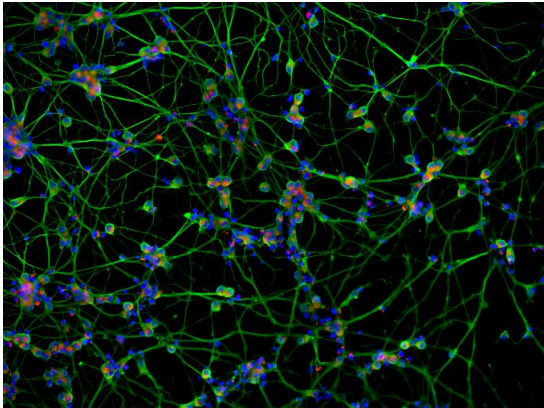

100 μm

## Supplementary Figure S2

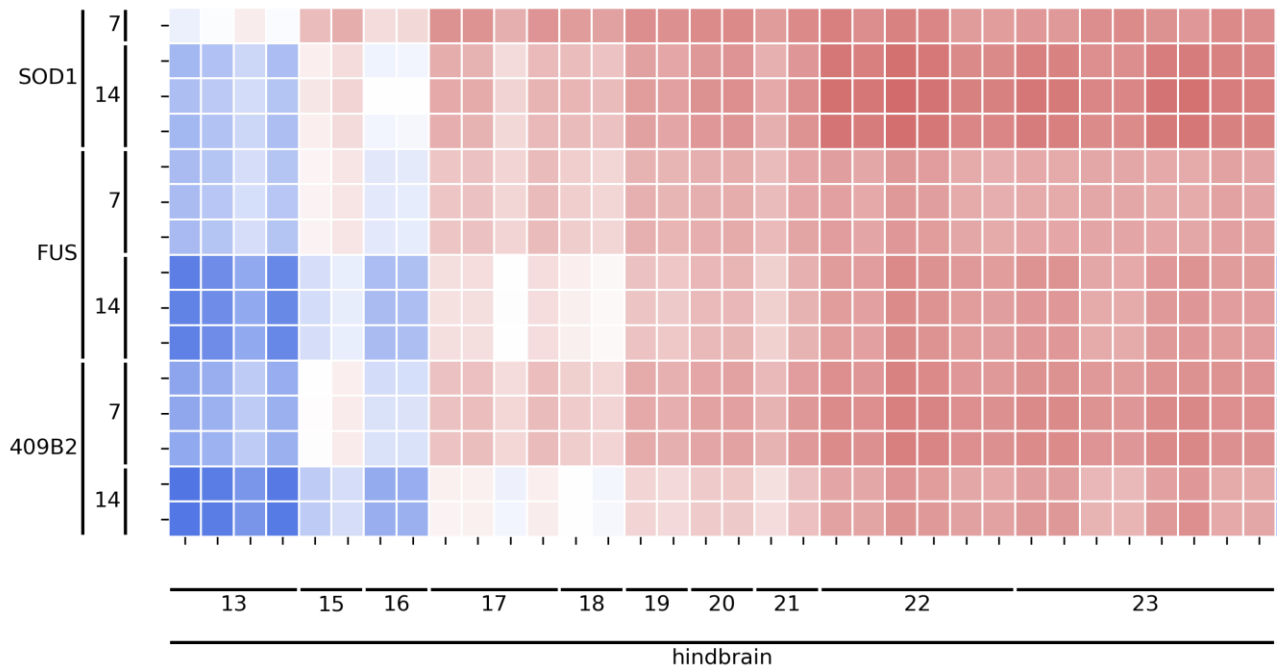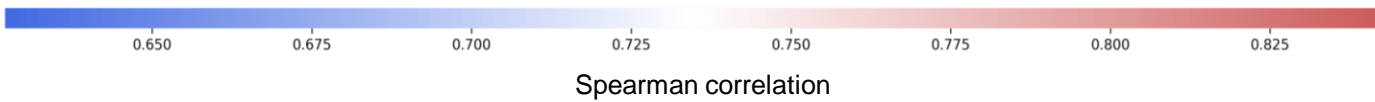

Supplement: Supplementary file 1 [file ijms-24-06987-s001.zip › Kondoet al SupFig_Submit_230407.pdf]
